# Evaluation of histopathological examinations of the cervix diagnosed as "other neoplasms" on the Cancer Information System, Brazil, 2013-2020: a descriptive study

**DOI:** 10.1590/S2237-96222022000300012

**Published:** 2022-12-02

**Authors:** Itamar Bento Claro, Mario Lucio Cordeiro Araújo, Caroline Madalena Ribeiro, Maria Beatriz Kneipp Dias, Jeane Tomazelli

**Affiliations:** 1 Instituto Nacional de Câncer José Alencar Gomes da Silva, Divisão de Detecção Precoce e Apoio a Organização de Rede, Rio de Janeiro, RJ, Brazil; 2 Instituto Nacional de Câncer José Alencar Gomes da Silva, Seção Integrada de Tecnologia em Citopatologia, Rio de Janeiro, RJ, Brazil; 3 Instituto Nacional de Câncer José Alencar Gomes da Silva, Divisão de Pesquisa Populacional, Rio de Janeiro, RJ, Brazil

**Keywords:** Uterine Cervical Neoplasms, Brazilian National Health System, Mass Screening, Access to Information, Epidemiology, Descriptive

## Abstract

**Objective:**

to describe and reclassify cervical histopathology test result diagnoses recorded as other neoplasms on the Cancer Information System (SISCAN), Brazil, 2013-2020.

**Methods:**

this was a descriptive study based on diagnoses input to the “other malign neoplasms” field on the SISCAN; a pathologist assessed the diagnoses and reclassified them based on the categories existing on the standardized record form; absolute and relative frequencies of incorrectly recorded diagnoses were calculated.

**Results:**

histopathology test results registered as “other malign neoplasms” accounted for 2.4% (n = 5,778) of all records, 67.4% of which in fact fell into categories already existing on the form, 8.9% were indeed other neoplasms and 24.5% were results not compatible with other neoplasms and were not covered by the form categories, such as benign findings or findings outside the cervix.

**Conclusion:**

the “other malignant neoplasms” field is frequently misused on the SISCAN; the analysis highlighted the need to train professionals to use the system properly, as well as the need to include new categories on the form.

**Study contributions**

**Main results**

Only 8.1% of diagnoses originally recorded as “other malign neoplasms” were classified correctly as such. 75.5% of the reclassified test results fell into diagnoses categories existing on the record form.

**Implications for services**

Pathology laboratories should train professionals to correctly use the form on the system and should monitor records input to the “other malign neoplasms” field.

**Perspectives**

The cervical histopathology test result form needs to be revised, to include diagnoses identified in this study that do not fall into the existing options.

## Introduction

Cervical cancer incidence remains high in Brazil, with an estimated annual occurrence of approximately 15 cases per 100,000 women, corresponding to 16,710 estimated new cases in 2022.^1^ This type of cancer is the third most frequent neoplasm in Brazilian women - excluding non-melanoma skin cancer - and there are marked regional differences in its occurrence. In 2020, Northern Brazil, with a standardized incidence rate of 9.5 cases per 100,000 women, was the region with the highest rate of cervical cancer, while in the Southeast region the incidence rate was approximately three times lower (3.4/100,000).^2^ ^-^ ^4^ The marked regional differences in incidence and mortality rates reflect the social inequities and inequalities between Brazil’s different regions with regard to social and economic development and access to health care.^3^ ^-^ ^5^

Brazil’s high cervical cancer mortality rates form a challenging scenario, and show that the established public policies have not yet had a positive impact on control actions and, consequently, on the mortality indicators for this disease.^6^ ^,^ ^7^ Problems such as screening program shortcomings, lack of a reminder system for the target population, inadequate follow-up of women with suspected or confirmatory cancer results, and insufficient quality control systems for cervical cancer cytopathology and histopathology tests are still very evident in Brazil.^8^

According to the Brazilian Guidelines for Cervical Cancer Screening (*Diretrizes Brasileiras do Rastreamento do Câncer do Colo do Útero*), investigation for diagnostic confirmation is necessary in the event of a screening test showing changes, either by repeating the cytopathology test or by performing colposcopy.^9^ Biopsy or excision of the lesion is indicated following colposcopy assessment, and final diagnosis is confirmed by histopathological analysis of the sample collected.^10^ Therapy is defined based on histopathological diagnosis, and the quality of this examination is important to avoid unnecessary procedures and to choose timely treatment.^11^

In Brazil, cervical histopathology tests performed by the National Health System (*Sistema Único de Saúde* - SUS) have been recorded on the Cancer Information System (*Sistema de Informação do Câncer* - SISCAN) since 2013. The SISCAN has a standardized form with pre-defined diagnosis options.^12^ In situations in which the result does not fit into the available options, the case is recorded in the “other malignant neoplasms” field of the system, and the type of neoplasm must be specified.

Distribution of histopathology test results is one of the indicators used to evaluate the performance of the cancer control program. Thus, it is expected that use of the “other malignant neoplasms” option, in relation to case diagnosis, should be infrequent, since the form contains options for the main histopathological diagnoses related to cervical cancer. However, only numeric fields are available for tabulation, and it is not possible, through the available tabulation tools, to identify what the “other neoplasms” are.

The objective of this study was to describe and reclassify cervical histopathology test result diagnoses recorded as other neoplasms on the SISCAN.

## Methods

### Design

This was a descriptive cross-sectional study of the information recorded on the Cancer Information System (SISCAN) “other malign neoplasms” field regarding cervical cancer histopathology test results between January 2013 and September 2020.

### Background

Histopathology tests are the method used to confirm cancer diagnosis. They are performed in pathology laboratories throughout the country. Test result reports are issued by pathologists and the results are determinant for the choice of treatment for each case. All histopathology tests for diagnostic investigation of cervical cancer performed by the SUS must be recorded on the SISCAN. 

The SISCAN is an open-access information system that has been in use since its introduction in 2013, with the purpose of enabling actions related to cervical cancer and breast cancer control to be monitored and, consequently, making it possible to standardize and improve the quality of mammography reports and records of cervix and breast cytopathology and histopathology tests performed within the SUS.^12^ ^,^ ^13^ Between 2013 and 2020, around 258,000 cervical histopathology examinations were recorded on the system.

On the histopathology test results form, the “other malign neoplasms” option refers to a category of tests with satisfactory results; however, diagnosis is not covered by any of the options available for information about lesions of a neoplastic or preneoplastic nature (Box 1).^12^ When the “other malign neoplasms” option is marked on the form, a description of the result found is required to be input to a field where it can be typed freely.

**Box 1:** Blocks of options for recording neoplastic or preneoplastic lesions found in cervical histopathology tests on the Brazilian Cancer Information System, Brazil

Benign lesions	Neoplastic or preneoplastic lesions		
	Block I	Block II	Block III
Squamous metaplasia	Cervical intraepithelial neoplasia grade I (CIN I)	Adenocarcinoma *in situ*	Other malignant neoplasms
Chronic nonspecific cervicitis	Cervical intraepithelial neoplasia grade II (CIN II)	Invasive adenocarcinoma	
Endocervical polyp	Cervical intraepithelial neoplasia grade III (CIN III)		
Cytoarchitectural changes with viral actions (HPV)^a^	Microinvasive epidermoid carcinoma		
	Invasive epidermoid carcinoma		
	Epidermoid carcinoma, invasion impossible to assess		

a) HPV: Human papillomavirus.

### Participants

The study included all cervical histopathology tests with results diagnosed as “other malign neoplasms” recorded on the SISCAN between 2013 and 2020.

### Variables

The following variables were selected:

- other malign neoplasms (open field);
- age (in years: up to 24; 25 to 64; 65 or over);
- type of surgical procedure (biopsy, conization, excision of the transformation zone, hysterectomy, other);
- region of Brazil in which the laboratory was located (North, Northeast, Midwest, South, Southeast).

### Data sources and measurement

The data were obtained from the SISCAN histopathology test database for the period from 2013 to 2020. This analysis period was defined considering the availability of the national consolidated database, accessed in January 2021.

Reclassification of diagnoses recorded as other neoplasms was performed by reviewing the diagnostic information recorded in the open field, without rereading the smear slide or anatomical specimen. The results were reclassified when the content described in this field corresponded to a diagnosis classification available on the SISCAN option list (Box 1). In situations in which it was not possible to reclassify the results into category options available on the form, the test results were categorized as “unspecified benign findings”, “benign findings outside the cervix”, “atypical glandular cells”, “inconclusive test”, “adenocarcinoma, invasion impossible to assess” and “other malign neoplasms outside the cervix”. Only results consistent with diagnosis as “other malignant neoplasms” remained classified as such. 

Following reclassification, we calculated the proportion of diagnoses incorrectly registered as “other malignant neoplasms”, the diagnosis of which corresponded to other options available on the system. 

### Statistical methods

The data held in the description field were extracted using the R software^14^ tydyverse package and then organized on Excel spreadsheets; the analysis was performed according to the macro-region in which the laboratory that issued the test report was located.

We calculated absolute and relative frequencies of the histopathology test results recorded on the SISCAN, according to age, laboratory macro-region and type of surgical procedure.

### Ethical aspects

The study project was approved by the Research Ethics Committee of the José Alencar Gomes da Silva National Cancer Institute (*Instituto Nacional de Câncer* - INCA), as per Opinion No. 3.007.666, issued on November 8, 2018, in accordance with Certificate of Submission for Ethical Appraisal No. 68203117.1.0000.5274.

## Results

Between 2013 and 2020, 248,497 histopathology tests were recorded on the SISCAN, 124,232 (50.5%) of which found results showing lesions of a preneoplastic nature, 2,051 (0.8%) of a neoplastic nature and 5,778 (2.4%) “other malign neoplasms” (Table 1).

**Table 1:** Distribution of cervical histopathology tests according to diagnosis result, Brazil, 2013-2020

Diagnosis result	n	%
Preneoplastic lesions	124,232	50.5
Neoplastic lesions	2,051	0.8
Other malign neoplasms	5,778	2.4
Benign or no findings	113,916	46.3
Total	245,977	100.0

Approximately 80% of the tests results classified as “other malignant neoplasms” were performed in the 25 to 64 years age group, within a case age spectrum ranging from 16 to 102 years. Biopsy (81.9%) and conization (9.9%) were the most frequent procedures of origin. Regarding regional distribution, Southern Brazil accounted for 36% of test results classified as other neoplasms (Table 2). 

**Table 2:** Characteristics of cervical histopathology tests diagnosed as “other malign neoplasms”, Brazil, 2013-2020

Variable	n	%
**Age (in years)**		
≤ 24	188	3.2
25-64	4,612	79.8
≥ 65	778	13.5
Unknown	200	3.5
**Type of surgical procedure**		
Biopsy	4,733	81.9
Conization	570	9.9
Excision of the transformation zone	165	2.9
Hysterectomy	229	3.9
Other	81	1.4
**Laboratory region**		
North	366	6.3
Northeast	1,154	20.0
Southeast	1,951	33.8
South	2,079	36.0
Midwest	228	3.9

After reviewing and reclassifying the findings recorded in the “other malign neoplasms” field, we found that 91.9% (n = 5,309) had been recorded incorrectly and only 8.1% (n = 469) were kept in this category. With regard to the reclassified results, 75.5% should have had the diagnosis indicated in the categories existing on the standardized form: 57.9% were diagnoses of lesions of a neoplastic nature, 17.3% were preneoplastic lesions, 12.6% were benign lesions, and 1.2% were unsatisfactory tests. 24.5% (n = 1,414) of the diagnoses recorded did not correspond to the classifications available on the form (Table 3).

**Table 3:** Diagnoses reclassified according to options existing on the form and not existing on the form (new classification), Brazil, 2013-2020

Variable	n	%	Per group (%)	Total (%)
**Neoplastic**			51.2	75.5
Other malign neoplasms	469	8.1		
Adenocarcinoma *in situ*	44	0.8		
Invasive adenocarcinoma	379	6.6		
Invasive epidermoid carcinoma	1,357	23.5		
Microinvasive epidermoid carcinoma	41	0.7		
Epidermoid carcinoma, invasion impossible to assess	664	11.5		
**Preneoplastic**			17.1	
CIN I^a^ (mild dysplasia)	75	1.3		
CIN II^b^ (moderate dysplasia)	20	0.3		
CIN III^c^ (severe dysplasia/carcinoma *in situ*)	897	15.5		
**Benign**			5.9	
Squamous metaplasia	18	0.3		
Chronic nonspecific cervicitis	191	3.3		
Cytoarchitectural changes with viral actions (HPV)^d^	88	1.5		
Endocervical polyp	49	0.8		
**Adequacy**			1.3	
Unsatisfactory test	72	1.3		
**Classifications not existing on the form**			6.6	24.5
**Benign**				
Unspecified benign findings	229	3.9		
Benign findings outside the cervix	143	2.5		
Atypical glandular cells	12	0.2		
**Adequacy**			11.0	
Inconclusive test	634	11.0		
**Neoplastic**			6.9	
Adenocarcinoma, invasion impossible to assess	156	2.7		
Other malign neoplasms outside the cervix	240	4.2		

a) CIN I: Cervical intraepithelial neoplasia grade I; b) CIN II: Cervical intraepithelial neoplasia grade II; c) CIN III: Cervical intraepithelial neoplasia grade III; d) HPV: Human papillomavirus.

In the analysis according to the Brazilian macro-regions, heterogeneity was found in the proportion of reclassified records, especially records of neoplastic and preneoplastic lesions. After reclassification, the proportion of “other malign neoplasms” results varied from 3.8% in the North to 16.2% in the Northeast, the latter being the region that continued to have the highest proportion of results classified as “other neoplasms” after analysis. (Table 4). In the period studied, invasive squamous cell carcinoma was the most frequent diagnosis in all macro-regions; except in the Southeast and Midwest, where it came in second position. In Brazil as a whole, invasive squamous cell carcinoma accounted for 23.5% of the reclassified diagnoses, while in the North, Northeast and South, these proportions were 38.3%, 32.5% and 22.3%, respectively. Cervical intraepithelial neoplasia (CIN) grade III was the second most frequent reclassification in the North and Southeast. In the Midwest, inconclusive test results accounted for the second most frequent classification.

**Table 4:** Diagnoses reclassified according to laboratory micro-region, Brazil, 2013-2020

Variable	Midwest			Northeast			North			Southeast			South			Brazil	
	n	%		n	%		n	%		n	%		n	%		n	%
**Neoplastic**																	
Other malign neoplasms	29	12.7		187	16.2		14	3.8		112	5.7		127	6.1		469	8.1
Adenocarcinoma *in situ*	1	0.4		9	0.8		1	0.3		17	0.9		16	0.8		44	0.8
Invasive adenocarcinoma	16	7.0		117	10.1		5	1.4		98	5.0		143	6.9		379	6.6
Invasive epidermoid carcinoma	39	17.1		375	32.5		140	38.3		339	17.4		464	22.3		1,357	23.5
Microinvasive epidermoid carcinoma	-	0.0		9	0.8		6	1.6		10	0.5		16	0.8		41	0.7
Epidermoid carcinoma, invasion impossible to assess	11	4.8		66	5.7		37	10.1		174	8.9		376	18.1		664	11.5
**Preneoplastic**																	
CIN I^a^ (mild dysplasia)	4	1.8		6	0.5		6	1.6		37	1.9		22	1.1		75	1.3
CIN II^b^ (moderate dysplasia)	1	0.4		3	0.3		2	0.5		7	0.4		7	0.3		20	0.3
CIN III^c^ (severe dysplasia/carcinoma *in situ*)	13	5.7		72	6.2		88	24.0		513	26.3		211	10.1		897	15.5
**Benign**																	
Squamous metaplasia	-	0.0		1	0.1		-	0.0		11	0.6		6	0.3		18	0.3
Chronic nonspecific cervicitis	2	0.9		7	0.6		12	3.3		91	4.7		79	3.8		191	3.3
Cytoarchitectural changes with viral actions (HPV)^d^	5	2.2		11	0.9		7	1.9		42	2.1		23	1.1		88	1.5
Endocervical polyp	4	1.8		13	1.1		1	0.3		12	0.6		19	0.9		49	0.8
**Adequacy**																	
Unsatisfactory test	2	0.9		10	0.9		6	1.6		33	1.7		21	1.0		72	1.2
**Classifications not existing on the form**																	
**Benign**																	
Unspecified benign findings	10	4.4		24	2.1		6	1.6		94	4.8		95	4.6		229	4.0
Benign findings outside the cervix	5	2.2		9	0.7		3	0.8		25	1.3		101	4.8		143	2.5
Atypical glandular cells	3	1.3		1	0.1		-	0.0		6	0.3		2	0.1		12	0.2
**Adequacy**																	
Inconclusive test	62	27.2		152	13.2		20	5.5		196	10.0		204	9.8		634	11.0
**Neoplastic**																	
Adenocarcinoma, invasion impossible to assess	9	3.9		32	2.8		2	0.6		41	2.1		72	3.5		156	2.7
Other malign neoplasms outside the cervix	12	5.3		51	4.4		10	2.8		93	4.8		74	3.6		240	4.2
Total	228	100.0		1,154	100.0		366	100.0		1,952	100.0		2,078	100.0		5,778	100.0

a) CIN I: Cervical intraepithelial neoplasia grade I; b) CIN II: Cervical intraepithelial neoplasia grade II; c) CIN III: Cervical intraepithelial neoplasia grade III; d) HPV: Human papillomavirus.

The proportion of neoplastic lesions after reclassification ranged from 38.4% in the Southeast to 66% in the Northeast, while the proportion of neoplastic lesions together with preneoplastic lesions ranged from 50% in the Midwest to 81.7% in the North.

## Discussion

In the present study we found that in the period from 2013 to 2020, the description of the findings of more than 90% of the results of cervical histopathology tests classified on the SISCAN as “other malign neoplasms” in fact was compatible with the classification categories predefined on the system. This finding indicates shortcomings that compromise the objectives of results standardization, such as achieving better communication between clinical professionals and surgeons, reducing misinterpretation and ambiguities, facilitating the description of diagnosis and monitoring data.^12^ ^,^ ^15^

Reclassification of the terms described under “other malignant neoplasms” made it possible to identify flaws in the filling in of the information and provided elements for the debate on the need to include new categories on the system’s standardized form, thus minimizing the heterogeneity of the histopathology reports issued.^15^

Diagnoses of “other malignant neoplasms outside the cervix” and “benign findings outside the cervix” accounted for almost 7% of the cases, and recording them is not provided for on the standardized form, which is intended exclusively for recording cervical cancer screening and diagnostic investigation procedures.^12^ However, it is possible that part of these cases came from cervical lesion biopsies, but when they were analyzed it was identified that the lesion originated from another organ. This finding may indicate the need to include a specific field for these situations on the system. 

The cervix biopsy histopathology test result is considered to be the gold standard for diagnosis, and is the basis for the clinical treatment procedure adopted by each professional.^16^ ^-^ ^18^ However, although histopathology tests are more accurate in detecting the concepts and standards adopted in the interpretation of smears,^16^ a literature review conducted in 2007 about quality control in cervical cytology highlighted the strong component of subjectivity found in histological analysis, which can result in high diagnostic variability.^19^

The reliability of conventional histopathology almost always depends on the knowledge and experience of the pathologist.^17^ Differentiation between benign and malignant lesions is based on the histopathological criteria described in the literature.^20^

Clinical procedure for treatment and prognosis depends on histopathology and the extent to which cancer has spread, i.e. the stage it is at. The histopathology test is an essential step, before more complex examinations are performed.^9^ For a more assertive diagnosis, it is important that tissue samples are of sufficient size and well preserved,^17^ which reinforces the importance of structuring screening test monitoring and quality control actions throughout the process.^18^ A previous study also indicated weakness in the classification of unsatisfactory histopathology tests, noting that 21% of tests had incorrectly informed diagnosis.^21^

It is noteworthy that when diagnosing “other neoplasms”, there are categories in which it is not possible to specify the type of neoplasm by morphology alone. In some cases, complementary studies are necessary, such as immunohistochemistry, to determine whether the lesion is primary or metastatic; and when primary, whether it is of squamous, glandular or mesenchymal origin.^22^

Inadequate input of diagnosis on the SISCAN also impacts use of data for monitoring and planning of control actions, and can compromise use of indicators based on diagnosis and limit comparisons with results from other programs.^23^ A lot of information, that should be in the comments field, is input as other neoplasms, such as the presence of glandular extension in squamous intraepithelial neoplasms. 

Monitoring cervical cancer control program indicators is fundamental in order to guide control actions, and there are several studies dedicated to evaluating the program’s performance and guiding the policy.^24^ ^,^ ^25^ Evaluation of the content recorded in the “other malignant neoplasms” field revealed problems in recording cervical cancer diagnosis that may lead to unnecessary interventions, as well as problems related to correct diagnosis. Monitoring of records with a high proportion of “other malignant neoplasms” by laboratories and health service managers may contribute to identifying points in the network that need improvement.^26^

One of the limitations of this study is that only one professional reviewed the data, making it impossible to assess discrepancies. However, many of the terms analyzed referred directly to diagnosis categories pre-defined on the system form. As such, it is expected that possible discrepancies were minimal. Another limitation of this study is the fact that the database examined does not contain all SUS test records performed, because the data we used refer only to services that have implemented the SISCAN. However, the findings point to (i) the need for regular monitoring of SISCAN information and (ii) raising the awareness of professionals as to the proper use of the system, considering that of the 5,778 exams recorded as other neoplasms, only 469 cases were correctly classified as such.

The fact that the study found that almost all (more than 90%) of the cervical histopathology test results recorded as “other malignant neoplasms” were incorrectly classified, highlights the need to intensify monitoring of information quality, with the aim of identifying possible biases responsible for the situation described.

We conclude that inadequate use of the “other malignant neoplasms” description field points to the need to train the professionals responsible for preparing test result reports, in addition to the need to adapt the Cancer Information System form to include new standardized categories.

## References

[1] Ministério da Saúde (BR), Instituto Nacional de Câncer José de Alencar Gomes da Silva (2020). Estimativa 2020. Incidência do Câncer no Brasil.

[2] Ministério da Saúde (BR), Instituto Nacional de Câncer José de Alencar Gomes da Silva (2022). Atlas de Mortalidade por Câncer.

[3] Girianelli VR, Gamarra CJ, Silva GA (2014). Os grandes contrastes na mortalidade por câncer do colo uterino e
de mama no Brasil. Rev Saude Publica.

[4] Tallon B, Monteiro D, Soares L, Rodrigues N, Morgado F (2020). Tendências da mortalidade por câncer de colo no Brasil em 5 anos
(2012-2016). Saude debate.

[5] Silva AG, Jardim BC, Ferreira VM, Junger WL, Girianelli VR (2020). Mortalidade por câncer nas capitais e no interior do Brasil: uma
análise de quatro décadas. Rev Saude Publica.

[6] Silva MJS, Bergmann A, Siqueira ASE, Casado L, Zamboni MM (2018). Influência das Iniquidades Sociais e dos Cuidados de Saúde na
Incidência e Mortalidade por Câncer. Rev Bras Cancerol.

[7] Nascimento MI, Massahud FC, Barbosa NG, Lopes CD, Rodrigues VC (2020). Mortalidade prematura por câncer de colo uterino: estudo de
séries temporais interrompidas. Rev Saude Publica.

[8] Claro IB, Lima LD, Almeida PF (2021). Diretrizes, estratégias de prevenção e rastreamento do câncer do
colo do útero: as experiências do Brasil e do Chile. Cien Saude Colet.

[9] Ministério da Saúde (BR), Instituto Nacional de Câncer José Alencar Gomes da Silva, Coordenação de Prevenção e Vigilância, Divisão de Detecção Precoce e Apoio a Organização de Rede (2016). Diretrizes brasileiras para o rastreamento do câncer do colo do
útero.

[10] Ministério da Saúde (BR), Instituto Nacional de Câncer José Alencar Gomes da Silva, Coordenação de Prevenção e Vigilância (Conprev) (2002). Falando sobre câncer do colo do útero.

[11] Pedrosa JHM, Lys PM (2011). Perfil das lesões encontradas nos histopatológicos do colo uterino em
pacientes com atipia de células glandulares.

[12] Ministério da Saúde (BR), Instituto Nacional de Câncer José de Alencar Gomes da Silva, Sistema de Informação do Câncer (2013). Manual preliminar para apoio à implantação.

[13] Brasil, Ministério da Saúde (2013). Portaria no 3.394, de 30 de dezembro de 2013. Institui o Sistema
de Informação de Câncer (SISCAN) no âmbito do Sistema Único de Saúde
(SUS).. Diário Oficial da União.

[14] R Foundation for Statistical Computing (2013). R Core Team. A Language and Environment for Statistical
Computing.

[15] Bacchi CE, Melo CRA, Franco MF, Artigiani R (2014). Manual de padronização de laudos histopatológicos.

[16] Ministério da Saúde (BR), Instituto Nacional de Câncer José Alencar Gomes da Silva, Coordenação de Prevenção e Vigilância, Divisão de Detecção Precoce e Apoio à Organização de Rede (2016). Manual de gestão da qualidade para laboratório de citopatologia.

[17] Stofler MECW, Nunes RD, Rojas PFB, Trapani A, Schneider IJC (2011). Avaliação do desempenho da citologia e colposcopia comparados com
a histopatologia no rastreamento e diagnóstico das lesões do colo
uterino. Arq Catarin Med.

[18] Al-Nafussi A, Colquhoun M (1990). Mild cervical intraepithelial neoplasia (CIN 1): a histological
overdiagnosis. Histopathology.

[19] Tavares SBN, Amaral RG, Manrique EJC, Sousa NLA, Albuquerque ZBP, Zeferino LC (2007). Controle da Qualidade em Citopatologia Cervical: Revisão de
Literatura. Rev Bras Cancerol.

[20] Ministério da Saúde (BR), Secretaria de Gestão do Trabalho e da Educação na Saúde, Departamento de Gestão da Educação na Saúde (2012). Caderno de referência 1: Citopatologia Ginecológica.

[21] Claro IB, Araújo MLC, Migowski A, Tomazelli JG (2021). Análise dos Motivos de Insatisfatoriedade dos Exames
Histopatológicos do Colo do Útero no Sistema Único de Saúde, Brasil, 2014 a
2017. Rev Bras Cancerol.

[22] Yaziji H, Gown AM (2001). Immunohistochemical analysis of gynecologic
tumors. Int J Gynecol Pathol.

[23] Laguardia J, Domingues CMA, Carvalho C, Lauerman CR, Macário E, Glatt R (2004). Sistema de informação de agravos de notificação em saúde (Sinan):
desafios no desenvolvimento de um sistema de informação em
saúde. Epidemiol Serv Saude.

[24] Santos RS, Melo ECP, Santos KM (2012). Análise espacial dos indicadores pactuados para o rastreamento do
câncer do colo do útero no Brasil. Texto Contexto Enferm.

[25] Costa RFA, Longatto-Filho A, Pinheiro C, Zeferino LC, Fregnani JH (2015). Historical Analysis of the Brazilian Cervical Cancer Screening
Program from 2006 to 2013: A Time for Reflection. PLoS ONE.

[26] Silva GA, Alcantara LLM, Tomazelli JG, Ribeiro CM, Girianelli VR, Santos EC (2022). Avaliação das ações de controle do câncer de colo do útero no
Brasil e regiões a partir dos dados registrados no Sistema Único de
Saúde. Cad Saude Publica.

